# *In vivo* longitudinal visualization of bone marrow engraftment process in mouse calvaria using two-photon microscopy

**DOI:** 10.1038/srep44097

**Published:** 2017-03-09

**Authors:** Viet-Hoan Le, Seunghun Lee, Seungwon Lee, Taejun Wang, Won Hyuk Jang, Yeoreum Yoon, Soonjae Kwon, Hyekang Kim, Seung-Woo Lee, Ki Hean Kim

**Affiliations:** 1 Division of Integrative Biosciences and Biotechnology, Pohang University of Science and Technology, 77 Cheongam-ro, Nam-gu, Pohang, Gyeongbuk 37673, Rep. of Korea; 2 Department of Mechanical Engineering, Pohang University of Science and Technology, 77 Cheongam-ro, Nam-gu, Pohang, Gyeongbuk 37673, Rep. of Korea; 3 Department of Life Sciences, Pohang University of Science and Technology, 77 Cheongam-ro, Nam-gu, Pohang, Gyeongbuk 37673, Rep. of Korea

## Abstract

Intravital microscopy of mouse calvarial bone marrow (BM) is a powerful method for studying hematopoietic stem cells (HSCs) and the BM microenvironment at the cellular level. However, the current method used to access the mouse calvaria allows for only a few imaging times in the same mouse because of scar formation and inflammation induced by multiple surgeries. Longitudinal imaging of the BM may help better understand its microenvironment. In this study, a mouse calvarial window model was developed for longitudinal imaging that involves attaching a cover glass window onto the mouse calvaria and sealing the surrounding exposed area with cyanoacrylate glue and dental cement. The model was used for the longitudinal two-photon microscopy (TPM) imaging of the BM engraftment process. The same BM cavity sites were imaged multiple times over 4 weeks after BM transplantation (BMT). Temporal changes in the BM microenvironment, such as the reconstitution of transplanted BM cells and the recovery of vasculature, were observed and analysed qualitatively and quantitatively. Longitudinal intravital microscopy using the mouse calvarial window model was successfully demonstrated and may be useful for further BM studies.

Hematopoietic stem cells (HSCs) are multipotent cells from which all blood cells, including those from myeloid and lymphoid lineages, are derived^1^,^2^. HSCs reside in the bone marrow (BM) niche, a specialized microenvironment that is required for the steady development of new blood cells and the self-renewal of HSCs^3^,^4^,^5^. The BM niche contains a multicellular complex that includes hematopoietic lineage cells, microvasculature with endothelial cells, osteolineage cells, and other stromal components^6^. The interaction between the BM compartments and HSCs is thought to play a critical role in maintaining and regulating HSCs^7^,^8^,^9^,^10^,^11^,^12^,^13^. Many pathological states are related to disturbances in the BM microenvironment^3^,^14^,^15^,^16^. Currently, an ideal method for studying the BM niche is intravital microscopy, which allows *in vivo* observation of the BM microenvironment at the single-cell level. Intravital microscopy has been conducted on mouse calvarial BM^17^,^18^,^19^,^20^,^21^,^22^,^23^, which is sufficiently thin and transparent for light penetration and high-resolution imaging. *In vivo* fluorescent microscopy of the mouse calvarial BM was first conducted by Irina B. Mazo *et al*. to study microvessels in the BM^24^. Technical advances in fluorescent microscopy led to the application of confocal microscopy and two-photon microscopy (TPM) in BM imaging. The use of both extrinsic labelling dyes and intrinsic contrasts, such as second-harmonic generation (SHG) from collagen and autofluorescence from cell cytoplasm^19^,^25^, in TPM enables the visualization of cells, vasculature, bone, and other compartments in the BM niche.

Intravital microscopy of mouse calvarial BM has been used in various studies on BM and its microenvironment. It has been used to monitor the distribution and behaviour of HSCs in mouse calvarial BM at the single-cell level^18^, and has revealed the regulation of HSCs through cell-to-cell interaction or regulator molecule-to-cell interaction^18^,^20^,^26^. In addition, studies of local oxygen tension^27^, BM vasculature, and BM endothelial cells^24^,^28^,^29^,^30^,^31^,^32^ have been conducted and HSCs have been tracked successfully the first few days after transplantation using intravital microscopy of mouse calvarial BM^18^,^33^. Intravital microscopy has been used to study molecular and cellular regulators of HSC homing^18^, and diabetic conditions was examined^34^. The interactions between BM-resident dendritic cells and mature B and T cells^35^,^36^, the behaviour of T and B cells in BM^37^,^38^,^39^, and the role of Treg cells in allo-HSC persistence were studied^40^. Finally, engraftment of tumour cells such as leukaemia or multiple myeloma in calvarial BM was tracked using intravital microscopy^14^,^29^,^41^.

Although intravital microscopy of mouse calvarial BM has enhanced our understanding of biological processes in BM, longitudinal intravital imaging of BM is still a challenge. Currently, to perform intravital microscopy of BM, a surgical procedure is needed to open the scalp skin to access the calvarial bone. The mouse scalp must be sutured after an imaging session and reopened for the next session of imaging the same calvarial BM. This procedure induces a scar on the bone surface that severely degrades the image quality. Therefore, it was impossible to image the same calvaria more than three or four times^25^,^42^. Moreover, repeated surgery before every imaging session could affect the health of the mouse and induce inflammation in the calvaria. In irradiated mice, surgery-induced trauma might interfere with recovery after BM transplantation (BMT) and should be considered. In addition, dye staining of BM stem cells leads to dilution of the dye via cell division, thus limiting the imaging time period^25^.

We developed a new surgical procedure for longitudinal BM imaging in which a glass window was implanted on the exposed calvarial bone for protection and imaging through. This mouse calvarial window model was used for a 4-week longitudinal observation of the engraftment process after BMT. The same BM sites were imaged multiple times over 4 weeks without the need for any additional surgery. Reconstitution of the transplanted BM cells and recovery of blood vessels in the calvarial BM were visualized and analysed.

## Results

### Mouse calvarial window model

**Figure 1:**
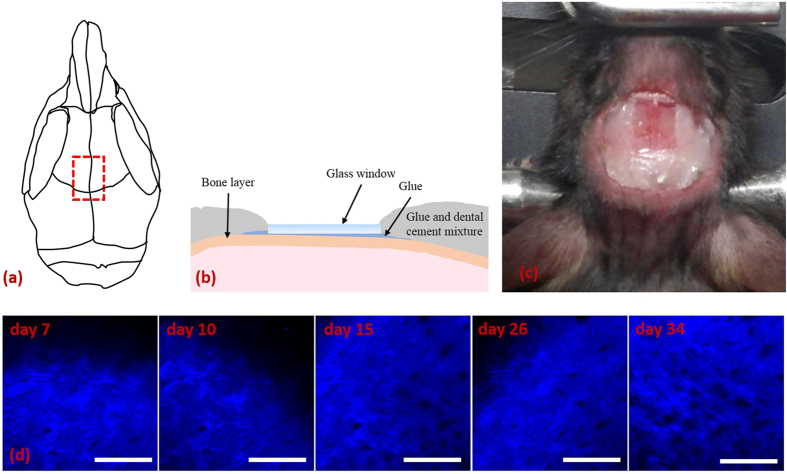
Mouse calvarial window model for longitudinal intravital BM microscopy. (**a**) Schematic of mouse calvaria on which the position of the glass window is indicated (red dashed box). (**b**) Schematic showing how the glass window is attached to the mouse skull with glue. (**c**) Photograph of the mouse calvarial window. (**d**) SHG images of the bone surface at the same site on the calvaria taken 7, 10, 15, 26, and 34 days after the glass window was attached. Scale bar: 100 μm.

The mouse calvarial window model was developed for the longitudinal imaging of mouse calvarial BM. Details of the model are given in the Methods section, but a brief description is provided here. Figure 1 presents schematics (Fig. 1(a,b)) and a photograph (Fig. 1(c)) of the window model and second-harmonic generation (SHG) images (Fig. 1(d)) of the same calvarial bone at different time points after the window was created. The entire procedure was conducted while the mouse was under gas anaesthesia. After the scalp was excised and removed to access the calvaria, a small amount of cyanoacrylate glue was applied and a small rectangular cover glass was attached directly onto the frontoparietal region of the calvaria (Fig. 1(a)). The surrounding exposed skull area was then covered with a mixture of dental cement powder and cyanoacrylate glue. A cross-sectional view of the glass window on the calvaria is shown in Fig. 1(b), and the sagittal suture in the skull is clearly visible through the glass window shown in Fig. 1(c). SHG images of the surface of the calvarial bone, taken 7, 10, 15, 26, and 34 days after cover glass attachment, were shown to examine scar formation (Fig. 1(d)). Collagen fibres and osteocyte-housing holes in the bone were clearly seen in the images at all time points. The quality of the SHG images confirmed that no scarring occurred on the bone surface. In addition, photographs of a BM calvarial window at different time points up to 34 days after cover glass attachment showed no indication of scar formation (Supplementary Fig. 1). There was no significant change in the maximum imaging depth in the photographs, which was approximately > 140 μm from the bone surface.

### TPM using the normal mouse calvarial window model

**Figure 2:**
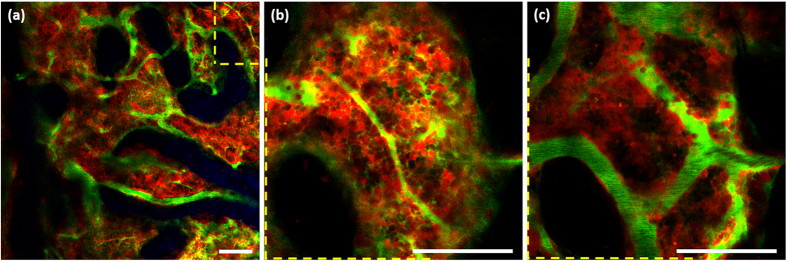
TP images of normal DsRed-expressing mouse calvarial BM cavity. (**a**) Large-FOV image at a depth of 80 μm (Supplementary Video 1); (**b**) small-FOV image at a depth of 80 μm (Supplementary Video 2); (**c**) small-FOV image at a depth of 100 μm. Blue: bone (SHG signal); red: DsRed-expressing cells; green: vasculature (FITC-dextran signal). Scale bar: 100 μm.

To demonstrate the feasibility of two-photon (TP) imaging using the mouse calvarial window model, the models made of DsRed-expressing mice (B6.Cg-Tg(CAG-DsRed*MST)1Nagy/J) were imaged 1 week after the glass window was attached. Three representative TP images of the mouse BM are shown in Fig. 2 (Supplementary Videos 1 and 2). One TP image had a large field of view (FOV) of 750 μm × 750 μm at a depth of 80 μm from the surface, and the other two TP images had a small FOV of 250 μm × 250 μm at depths of 80 and 100 μm from the surface. DsRed-expressing cells, blood vessels containing fluorescein isothiocyanate (FITC)-dextran, and bones intrinsically expressing SHG appeared as red, green, and blue, respectively. The large-FOV image shows the distribution of BM cavities inside the mouse calvaria and that the cavities are interconnected and occupy more than 50% of the area (Fig. 2(a)). The small-FOV images are enlarged images of the yellow dashed-line region on the large-FOV image and show the distribution of cells and blood vessels in the cavity. At a depth of 80 μm from the surface, the cavity was full of interleaved blood vessels and cells, most of which were red by expressing DsRed. However, there were some cells not expressing DsRed but were sparsely visible. Dispersed green signals seen near some vessels in Fig. 2(b) may have been due to leakage of FITC-dextran through the vessel walls. At a depth of 100 μm from the surface, DsRed-expressing cells were densely distributed along the large blood vessels that contained slowly flowing or migrating cells (Fig. 2(c)). Time-lapse image of a different BM cavity (Supplementary Video 3) shows cells migrating along the wall in the large blood vessels. Most cells in the cavity were relatively stationary during imaging time.

### Longitudinal TPM of the BM engraftment process

**Figure 3:**
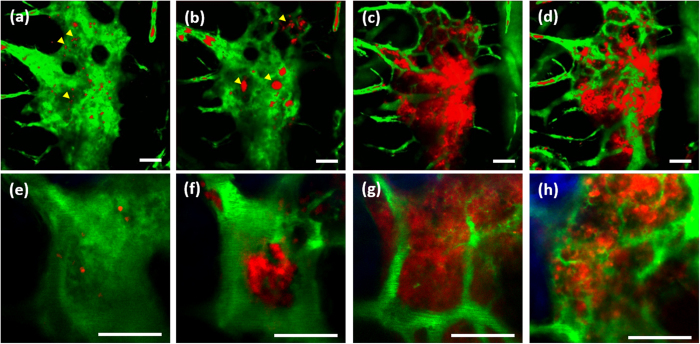
Longitudinal TP images of the same BM cavity post BMT. (**a–d**) MIP TP images in a large FOV on days 1, 4, 9, and 20 post BMT, respectively (Supplementary Videos 4,5,6,7). (**e–h**) Single-plane TP images in a small FOV 110 μm below the bone surface on days 1, 4, 9, and 20 post BMT, respectively (Supplementary Videos 8,9,10,11). Blue: bone (SHG signal); red: transplanted BM cells (DsRed signal); green: vasculature (FITC-dextran signal). Scale bar: 100 μm.

The mouse calvarial window model was used to do longitudinal TP imaging of the BM engraftment process for 4 weeks after a lethal dose irradiation and subsequent BMT. In order to visualize the reconstitution of transplanted BM cells, DsRed-expressing BM cells were transplanted into C57BL/6 mouse calvarial window models. Longitudinal TP images of the same BM cavity sites obtained on days 1, 4, 9, and 20 post BMT are shown in Fig. 3 (Supplementary Videos 4,5,6,7,8,9,10,11). Maximum intensity projection (MIP) images at a large FOV are shown in Fig. 3(a–d) to show the general changes in the BM. TP images of a small FOV at a single depth are shown in Fig. 3(e–h) to show detail at the single-cell level.

The large- and small-FOV TP images taken on day 1 post BMT (Fig. 3(a,e)) show the entire cavity was filled with FITC-dextran, which had been injected intravenously, because of leakage from blood vessels damaged by irradiation. These images also show sparsely distributed red spots, which were either single or cluster of transplanted cells, indicating the occurrence of homing in the BM. The small-FOV TP image obtained on day 1 post BMT shows many negative-contrast cells in the cavity; these cells may be recipient BM cells still in the cavity. Both volumetric (Supplementary Video 8) and time-lapse TP images (Supplementary Video 12) show flows across the entire cavity because of disruption of blood vessels.

The large- and small-FOV TP images taken on day 4 post BMT (Fig. 3(b,f)) show clusters of transplanted DsRed-expressing BM cells and some blood vessels. The large-FOV TP image (Fig. 3(b)) shows several clusters of transplanted cells in the cavity. These cell clusters are much bigger than those seen on day 1 post BMT, indicating that the transplanted cells have been amplified. There are fewer of them compared to the number seen on day 1 post BMT. Some large cell clusters are at different locations from where the small clusters were seen on day 1 post BMT (Fig. 3(a)), which might be indicative of active migration of the transplanted cells in the early stage of the BM engraftment process. Most of the cavity space was still filled with FITC-dextran from leakage. The blood vessels partially visible in the upper right corner of the image were the result of the recovery of the damaged endothelial cells. The small-FOV TP image taken on day 4 post BMT (Fig. 3(f)) shows a large cluster of transplanted cells in the middle of the cavity. This cell cluster might have grown from a small cluster at the same location on day 1 post BMT. The number of cells clearly increased between days 1 and 4 post BMT. Because the transplanted BM cells were densely packed in the cluster, it was difficult to resolve individual cells even in the small FOV. There were fewer recipient cells, which show as negative contrast in the cavity, than on day 1 post BMT. Partially recovered blood vessels are seen in the upper right corner, and a small blood vessel is seen going through the cell cluster. Except for these blood vessels, most of the cavity was filled with leaked FITC-dextran.

TP images taken on day 9 post BMT (Fig. 3(c,g)) show largely expanded transplanted BM cells and a blood vessel network in the cavity. The large-FOV TP image (Fig. 3(c)) shows a dramatic expansion of the transplanted BM cells, indicating continued amplification. In addition, they appear to be more densely packed than in the control case (Fig. 2). The network of blood vessels was clearly visible owing to the retention of FITC-dextran in the vessels. The small-FOV TP image (Fig. 3(g)) shows further recovery of the transplanted BM cell population and the blood vessel network compared to that seen on day 4 post BMT. Recipient cells, which appear as negative contrast in the TP images of days 1 and 4 post BMT, are not easily detectable. Thick blood vessels surrounding the cavity and thin blood vessels in the middle of the cavity are clearly seen (Fig. 3(g)).

TP images of mouse calvarial BM taken on day 20 post BMT (Fig. 3(d,h)) show further reconstitution of the transplanted BM cells and vasculature. The large-FOV TP image (Fig. 3(d)) shows that the transplanted BM cells are less densely packed than on day 9 post BMT. The density of the BM cells was comparable to that in normal mice (Fig. 2), indicating that the BM population might have gone into the steady state. The vascular network was denser than on day 9 post BMT, probably because of continued recovery and development. The small-FOV TP image (Fig. 3(h)) shows changes in the transplanted BM cells and blood vessels similar to those seen in the large-FOV image. Because of the reduction in cell density, individual cells can be resolved in this small-FOV TP image. The DsRed-expressing cells flowing in the blood vessels indicate the presence of transplanted BM cells in the circulatory system. Further imaging on day 28 post BMT (Supplementary Fig. 2) showed results closely similar to those on day 20 post BMT, confirming that the BM cavity had fully recovered and reached the steady state.

### Time-lapse imaging during the BM engraftment process

**Figure 4:**
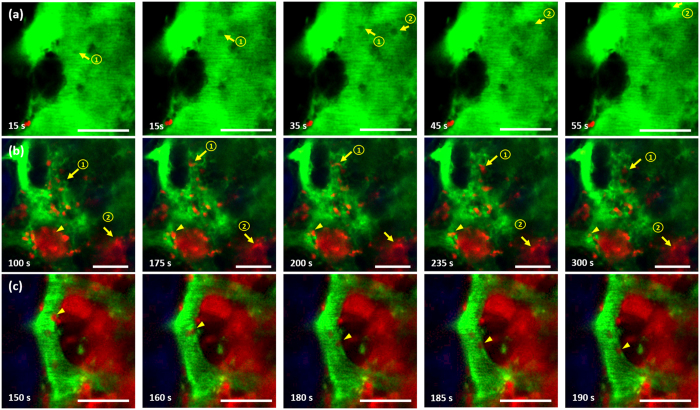
Time-lapse TP images of a BM cavity on (**a**) day 4 post BMT (Supplementary Video 13), (**b**) day 9 post BMT (Supplementary Video 14), and (**c**) day 13 post BMT (Supplementary Video 15). Yellow arrows and arrowheads track cell movement. Blue: bone (SHG signal); red: transplanted BM cells (DsRed signal); green: vasculature (FITC-dextran signal). Scale bar: 50 μm.

In addition to volumetric imaging, time-lapse imaging was performed as part of the longitudinal imaging of the BM engraftment process to observe dynamic cell behaviour in the BM. Images were taken at fixed depths inside the cavity at an imaging speed of 0.2 frames per second (fps), the same as that used for volumetric imaging. Each session lasted 350 s. Representative time-lapse TP images of a BM cavity taken on days 4, 9, and 13 post BMT are shown in Fig. 4(a–c) (Supplementary Videos 13,14,15) respectively. TP images of this cavity site show relatively delayed engraftment compared to that seen in Fig. 3. Time-lapse images taken on day 4 post BMT (Fig. 4(a)) show cell motility as the transplanted cells were forming small clusters. Transplanted DsRed-expressing BM cells were mostly stationary during imaging, and some recipient cells migrated in the direction of flow (numbered yellow arrows in Fig. 4(a)). Time-lapse images taken on day 9 post BMT (Fig. 4(b)) show cell motility as the transplanted BM cells expanded and partially filled the cavity. Some BM cells migrated across the cavity (numbered yellow arrows in Fig. 4(b)) or the blood vessels (yellow arrowhead in Fig. 4(b)). Shape change of the migrating cells was observed. Most of the other cells remained stationary during the imaging session. Time-lapse images taken on day 13 post BMT (Fig. 4(c)) show cell motility as the transplanted BM cells occupied most of the cavity. The network of blood vessels is also clearly visible. Some transplanted BM cells moved inside the blood vessels along the vessel wall (yellow arrowhead in Fig. 4(c)). Although this time-lapse imaging was conducted at a relatively slow speed and within a limited period, some dynamic behaviours of the transplanted BM cells were captured at various stages of BM engraftment.

### Analysis of vasculature recovery during the BM engraftment process

**Figure 5:**
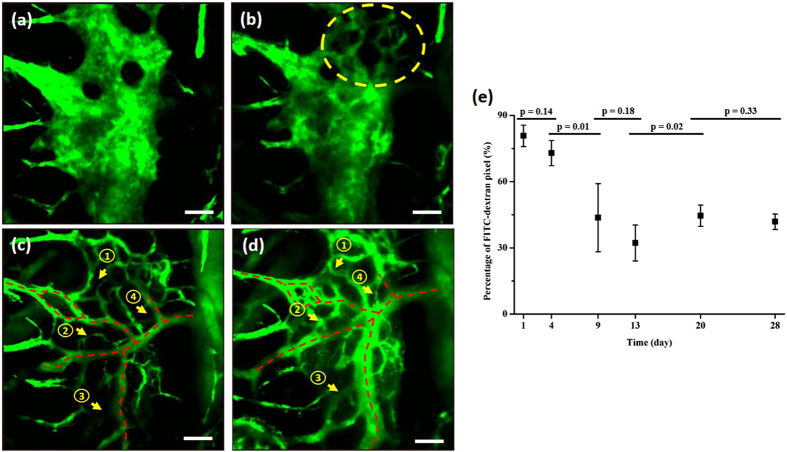
MIP TP images of BM vasculature on (**a**) day 1, (**b**) day 4, (**c**) day 9, and (**d**) day 20 post BMT. Yellow dashed oval in (**b**) indicates a region of vasculature recovery on day 4. Red dashed lines and numbered yellow arrows in (**c**) and (d) indicate the same large and small blood vessels, respectively, on days 9 and 20 post BMT. (e) Percentage of FITC-dextran (in pixels) in the BM cavities from day 1 to day 28 post BMT. Statistical analysis included two-tailed type-2 *t*-test; *p* ≤ 0.05 was considered statistically significant. Scale bar: 100 μm.

Recovery of vasculature after BMT was analysed by processing longitudinal three-dimensional (3D) large-FOV TP images. Vasculature images were obtained by extracting FITC-dextran channel images from the multichannel volumetric TP images and projecting them onto a plane using MIP. MIP vasculature images on days 1, 4, 9, and 20 post BMT are shown in Fig. 5(a–d), respectively. Some major blood vessels are marked with red dashed lines to track changes. The MIP vasculature image taken on day 1 post BMT (Fig. 5(a)) shows that the BM cavity was filled with FITC-dextran owing to blood vessel damage caused by irradiation. On day 4 post BMT, most of the cavity was still filled with leaked FITC-dextran, but some blood vessels were visible (area in yellow dashed oval in Fig. 5(b)), indicating partial recovery. The MIP vasculature image taken on day 9 post BMT (Fig. 5(c)) shows a clear view of the vascular network owing to complete retention of FITC-dextran in the blood vessels, some of which were thin and discontinuous. Some blood vessels were thicker on day 20 post BMT than on day 9 post BMT (yellow arrow 2 in Fig. 5(c,d)), indicating an increase in the lumen diameter of the vessel. In addition, there were newly formed microvessels on day 20 (yellow arrows 1, 3, and 4 in Fig. 5(c,d)). To quantify the vasculature recovery, the volumetric ratio of FITC-dextran (in pixels) in the BM cavities at various time points was calculated and presented in Fig. 5(e). On days 1 and 4 post BMT, the volumetric ratios were high because of leakage, and on days 9 and 13, it decreased because the leakage had stopped. Then, the ratio increased slightly but significantly (*p* = 0.02) on day 20 and fluctuated on day 28 post BMT (*p* = 0.33).

### Reconstitution of transplanted BM cells

**Figure 6:**
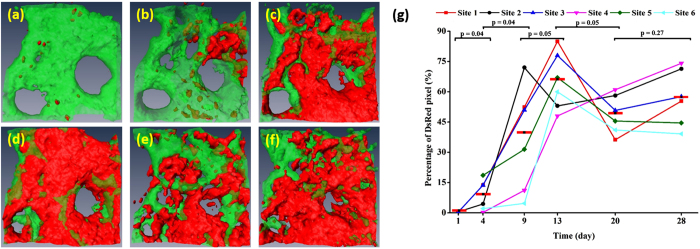
3D TP iso-surface images of a BM cavity site at different time points post BMT and a graph showing temporal changes of transplanted cell volumes at different cavity sites. (**a–f**) 3D TP iso-surface images on days 1, 4, 9, 13, 20, and 28 post BMT, respectively. Red: transplanted BM cells; green: vasculature. (**g**) Percentage of DsRed-expressing cells (in pixels) at six different BM cavity sites from days 1 to 28 post BMT (sites 4, 5, and 6 were not imaged on day 1). Mean values are indicated by the red horizontal bars. Statistical analysis included a two-tailed type-2 *t*-test; *p* ≤ 0.05 was considered statistically significant.

Reconstitution of the transplanted BM cells was analysed quantitatively by calculating the volumetric ratio of transplanted DsRed-expressing cells to the BM cavity volume as a function of time; the results are shown in Fig. 6. Longitudinal 3D isosurface images of a BM cavity on days 1, 4, 9, 13, 20, and 28 post BMT are shown in Fig. 6(a–f), respectively, and plots of the temporal change in the volumetric ratio of transplanted DsRed-expressing cells at six different cavity sites of five mouse models are shown in Fig. 6(g). The isosurface images show that the cell population increased up to day 13 post BMT and then decreased and fluctuated. Temporal changes in the volumetric ratio at multiple sites are indicative of the quantitative changes in the cell population, which increased relatively slowly between days 1 and 4 and then rapidly between days 4 and 13 post BMT. After peaking, the cell population fluctuated on days 20 and 28 post BMT. Statistical analysis with a 95% confidence interval showed that the changes in the BM cell population between adjacent imaging time points were significant until day 20 post BMT and not significant between days 20 and 28 post BMT. Variations in the evolution of the cell population, which depended on the cavity sites, were probably due to the heterogeneity of the engraftment process rate.

## Discussion

Although intravital microscopy is currently the least invasive method for *in vivo* imaging of the mouse calvarial BM niche and its compartments, performing long-term and repeated observations of the BM has been difficult because of the inflammation and scar formation caused by repeated scalp-opening surgery. In this study, we developed the mouse calvarial window model and demonstrated longitudinal intravital microscopy of mouse calvarial BM by visualizing the engraftment process of transplanted BM cells for approximately 4 weeks. Homing and repopulation of BM cells and the recovery of damaged vasculature were observed and the dynamic behaviour of transplanted BM cells during the BM engraftment process was monitored. The mouse calvarial window model requires only one scalp-opening surgery to attach a glass window onto the calvaria. Surgery is performed 1 week before the first imaging session to let any inflammation subside and reduce stress on the mouse. The small glass window allows the same cavity sites to be easily located for multiple imaging sessions. Previous studies tracked individual HSCs up to 2 days after BMT with the use of lipophilic cyanine dyes to label the BM stem cells^18^,^25^. In this study, DsRed-expressing BM cells, not diluted with labelling dyes, were transplanted into the BM for long-term observation.

TPM observations of the BM engraftment process at the same cavity sites revealed precisely the temporal evolution of the transplanted BM cells and the blood vessels. On day 1 post BMT, the donor BM cells homed to the recipient BM cavities by forming small cell clusters, which was consistent with the findings of a previous study^18^. The cell population increased until day 9 or 13 post BMT and then decreased to a steady-state level by day 20 post BMT. Why the BM cell population peaked on day 9 or 13 post BMT may be explained as follows: Immediately after irradiation, the immune system of the recipient mouse was extensively damaged; therefore, the transplanted BM cells needed to rapidly differentiate into the functional blood cells, resulting in a high BM cell population. As recovery progressed, the need for blood cells decreased and thus the BM cell population decreased. In addition, the engraftment process was asynchronous and depended on the BM cavity site, i.e., it started on day 1 post BMT at some sites and on day 4 post BMT at other sites (sites 4 and 6 in Fig. 6(g)). This may indicate that there are two engraftment types: an early primary engraftment resulting from the homing and differentiation of donor BM cells, and a late secondary engraftment resulting from the migration of differentiated donor BM cells at other BM cavity sites.

In the current study, time-lapse imaging clearly captured the dynamic behaviour of the BM cells. Some BM cells actively migrated during the expansion stage and might be active immune cells related to the immune response after irradiation. Some BM cells adhered to and rolled on the walls of blood vessels, indicating possible interaction between BM cells and endothelial cells, which was described in a previous study^33^.

The recovery of the vascular network in the BM also was imaged and analysed in detail in this study. Images from days 1 and 4 post BMT showed that FITC-dextran had leaked into the entire cavity because of the damage to the endothelial cells by irradiation, resulted in the increased permeability of the BM vessels to small-sized particles. This leakage was consistent with observations made in previous studies^18^,^19^. The leakage of FITC-dextran through the vessel wall stopped by day 9 post BMT, indicating that the vasculature and endothelial cells had recovered. This result was consistent with that of a previous study on sinusoidal endothelial cells^26^.

Although this study was successful in showing the entire BM engraftment process over the 4 weeks following BMT, there were some limitations. First, although attaching the glass window to the mouse calvaria removed the need for multiple surgeries for a longitudinal study, and thus their negative effects, the repeated injection of FITC-dextran may cause some stress to the mouse. Blood vessel imaging techniques based on intrinsic contrast, e.g., optical coherence tomography angiography, could be used in the future to overcome this problem. Second, for longitudinal imaging, the transplanted BM cells were DsRed-expressing cells and it was not possible to distinguish between the different cell types after the cell differentiation. Transferring cells that express different fluorescent proteins could resolve this problem. Finally, the optical properties of the glass window degraded over time owing to scratches on the surface of the glass. The use of adhesive covers to protect the glass can help prevent scratches and reduce degradation.

In conclusion, the newly developed mouse calvarial window model was used with two-photon microscopy to image the long-term BM engraftment process for the first time. The results provide additional information about the BM engraftment process and contribute to the optimisation of BMT. Furthermore, the mouse calvarial window model may be helpful in other longitudinal calvarial BM imaging studies.

## Methods

### Implementation of mouse calvarial window model

All animal experiments complied with the guidelines and regulations of the POSTECH Institutional Animal Care and Use Committee (IACUC; approval No. POSTECH-2015-0030-R1). The mouse calvarial window was created in 9-week-old female wild-type C57BL/6 mice and 10-week-old female DsRed-expressing mice [B6.Cg-Tg(CAG-DsRed*MST)1Nagy/J]. Before each imaging session, the mice were kept under controlled conditions at the POSTECH Biotech Center.

One week before the first imaging session, the calvarial window was created by attaching a cover glass on the mouse calvaria as follows: First, the mice were anaesthetised with 1.5%/vol isoflurane (Terrell™, Piramal Critical Care, Mumbai, India) and medical grade oxygen and placed on a heating plate (Chamlide TR, Live Cell Instrument, Seoul, Korea). After removing the scalp hair with a veterinary hair clipper, an incision was made through the scalp and an approximately 1 cm × 1 cm area of scalp tissue was removed. The calvarial bone was cleaned thoroughly by flushing it with phosphate-buffered saline and wiping it with sterile gauze until all the hair fragments and the connective membrane layer were removed. A 4 mm × 2 mm × 0.17 mm cover glass was carefully attached directly over the frontoparietal region of the calvarial bone using cyanoacrylate glue (Loctite 401, Henkel Corp.) (Fig. 1(a,b)). Cyanoacrylate glue was used because it is biocompatible and transparent when dried and it can prevent the regrowth of the membrane layer^43^. It is important to ensure that there are no bubbles in the glue layer under the glass window because they can cause optically opaque regions. The exposed calvarial bone area around the cover glass was covered with a layer of a mixture of cyanoacrylate glue and dental cement powder. This layer was made high enough to keep the immersion water from running out of the imaging region. After the glue dried, povidone-iodine was applied to the surrounding area to prevent infection caused by the surgical incision. A photograph of the mouse calvarial window is presented in Fig. 1(c).

### Bone marrow transplantation

Wild-type C57BL/6 mice were the recipient mice and DsRed-expressing mice were the donor mice. One week after the window was attached, the recipient mice were irradiated with two 4.8 Gy doses administered 4 h apart. The 10-week-old donor DsRed-expressing mice were sacrificed and whole BM cells were harvested by crushing the femur BM. 4 × 10^6^ total BM cells were transplanted into each recipient mouse by intravenous injection 24 h after irradiation. One week before and 2 weeks after the BMT, the recipient mice were fed antibiotic-containing water (ampicillin 1 g/l; Thermo Fisher Scientific).

### Two-photon microscopy

Two DsRed-expressing mice that underwent the mouse calvarial window procedure were used for TP imaging of normal BM. For BM transplanted mice, the same calvarial BM cavity sites of five BM transplanted mice with the calvarial window underwent longitudinal TPM imaging on days 1, 4, 9, 13, 20, and 28 post BMT. Mice were imaged at the same circadian time on each imaging day to avoid any adverse effects from a difference in circadian time. Before TP imaging, the mice were administered an intravenous injection of 80 μl of 50 mg/ml fluorescein isothiocyanate (FITC) dextran (FD2000S; Sigma-Aldrich, St. Louis, MO, USA) to label the BM vasculature. They were then anaesthetised with a mixture of 1.5%/vol isoflurane and medical grade oxygen. The mouse heads were stabilised with a stereotactic holder during the imaging session to eliminate motion artefacts. A temperature-controlled heating plate (Chamlide TR) kept the body temperature of the mice at 37 °C. Before imaging, the calvarial window was cleaned with a methanol swab.

All TP imaging was conducted using a commercial two-photon microscope (TCS SP5 II MP, Leica, Wetzlar, Germany) equipped with a Ti:sapphire laser (Chameleon Vision II, Coherent Inc., Santa Clara, CA, USA) with a 140-fs pulse width and 80-MHz repetition rate. Because we used one excitation wavelength of 920 nm to excite both DsRed and FITC-dextran simultaneously, no sequential imaging was needed. At this excitation wavelength, the wavelength of the SHG signal was near 460 nm. A 20× objective lens (HCX APO L20 × 1.0NA, water immersion, Leica) with a stepwise increment of 2 μm was used to obtain the 3D TP images. A set of dichroic mirrors and band-pass filters spectrally resolved the SHG, FITC-dextran, and DsRed emission signals to three channels of 430-480 nm, 500-550 nm, and 565-675 nm, respectively. Photomultiplier tubes (R10467U-40, Hamamatsu, Japan) then detected the signals. The images were 512 pixels × 512 pixels, the area of the large-FOV images was 775 μm × 775 μm, and the area for the small-FOV images was 258 μm × 258 μm. The laser power at the back aperture of the objective lens was in the range of 25-100 mW and compensated for during each imaging stack. The imaging speed was 0.2 fps. For time-lapse imaging, 70 frames were obtained at a single depth that was *x*-*y* scanned at 0.2 fps. For each mouse, after focusing the laser onto bone surface, a live, fast and large-FOV scanning was performed to find some main features of the BM cavity, such as the centre sinus and large blood vessels. Based on the relative position of the cavity with respect to the centre sinus, the shape of the cavity, and the large blood vessel network, the same cavities were located.

### Image processing and analysis

The acquired TP data were post-processed to increase contrast and visibility and then underwent quantitative analysis. Both image processing and analysis were performed using Leica Application Suite software (LAS AF Lite, Leica), Amira software (Amira 5.3.3, Visage Imaging, Richmond, VIC, Australia), and MATLAB (Matlab R2015a, MathWorks, Natick, MA, USA). TP images were presented in pseudocolours of blue (430-480 nm) for SHG from bone collagen, green (500-550 nm) for fluorescence from FITC-dextran, and red (565–675 nm) for fluorescence from the DsRed signal. For large-FOV TP and vasculature images, MIP images were generated and used as representative images. Videos were made of volumetric images to play at 5 fps and of time-lapse images to play at 8 fps.

Three-dimensional isosurface images of FITC-dextran and DsRed channels were rendered to facilitate assessment of the cell population. In addition, the volumetric ratio of the FITC-dextran signal and of the transplanted DsRed-expressing cells were calculated as the percentage of the number of FITC-dextran pixels and the number of DsRed pixels with respect to the total number of BM cavity pixels as follows:


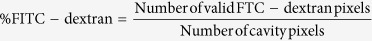



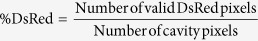


For each small-FOV (258 μm × 258 μm) TP image stack, the volume analysed was 60 μm below the starting depth of the BM cavity. A noise threshold was set to determine the valid FITC-dextran pixels from the FITC-dextran channel and the valid DsRed pixels from the DsRed channel. Cavity pixels included DsRed pixels, FITC-dextran pixels, and no-signal pixels among them. The calculation algorithm is presented in more detail in Supplementary Fig. 3. A two-tailed type-2 *t*-test was used for statistical analysis and *p* ≤ 0.05 was considered statistically significant.

## Additional Information

**How to cite this article:** Le, V.-H. *et al. In vivo* longitudinal visualization of bone marrow engraftment process in mouse calvaria using two-photon microscopy. *Sci. Rep.* **7**, 44097; doi: 10.1038/srep44097 (2017).

**Publisher's note:** Springer Nature remains neutral with regard to jurisdictional claims in published maps and institutional affiliations.

## Supplementary Material

Supplementary Information

Supplementary Video 1

Supplementary Video 2

Supplementary Video 3

Supplementary Video 4

Supplementary Video 5

Supplementary Video 6

Supplementary Video 7

Supplementary Video 8

Supplementary Video 9

Supplementary Video 10

Supplementary Video 11

Supplementary Video 12

Supplementary Video 13

Supplementary Video 14

Supplementary Video 15
