# Supplementary material for: In vivo longitudinal visualization of bone marrow engraftment process in mouse calvaria using two-photon microscopy
Source: Sci Rep. 2017 Mar 9;7:44097. doi: 10.1038/srep44097 (PMC5343427; doi:10.1038/srep44097)
Supplement: Supplementary Information [file srep44097-s1.pdf]

Supplementary Information

In vivo longitudinal visualization of bone marrow engraftment process in mouse calvaria using two-photon microscopy

Viet-Hoan Le[1], Seunghun Lee[2], Seungwon Lee[1], Taejun Wang[1], Won Hyuk Jang[1], Yeoreum Yoon[2], Soonjae Kwon[2], Hyekang Kim[1], Seung-Woo Lee[1,3,*], Ki Hean Kim[1,2,*]

Affiliations:

[1]Division of Integrative Biosciences and Biotechnology, Pohang University of Science and Technology, 77 Cheongam-ro, Nam-gu, Pohang, Gyeongbuk 37673, Rep. of Korea

[2]Department of Mechanical Engineering, Pohang University of Science and Technology, 77 Cheongam-ro, Nam-gu, Pohang, Gyeongbuk 37673, Rep. of Korea

[3]Department of Life Sciences, Pohang University of Science and Technology, 77 Cheongam-ro, Nam-gu, Pohang, Gyeongbuk 37673, Rep. of Korea

*corresponding author: sw_lee@postech.ac.kr, kiheankim@postech.ac.kr

Supplementary Figures & Legends

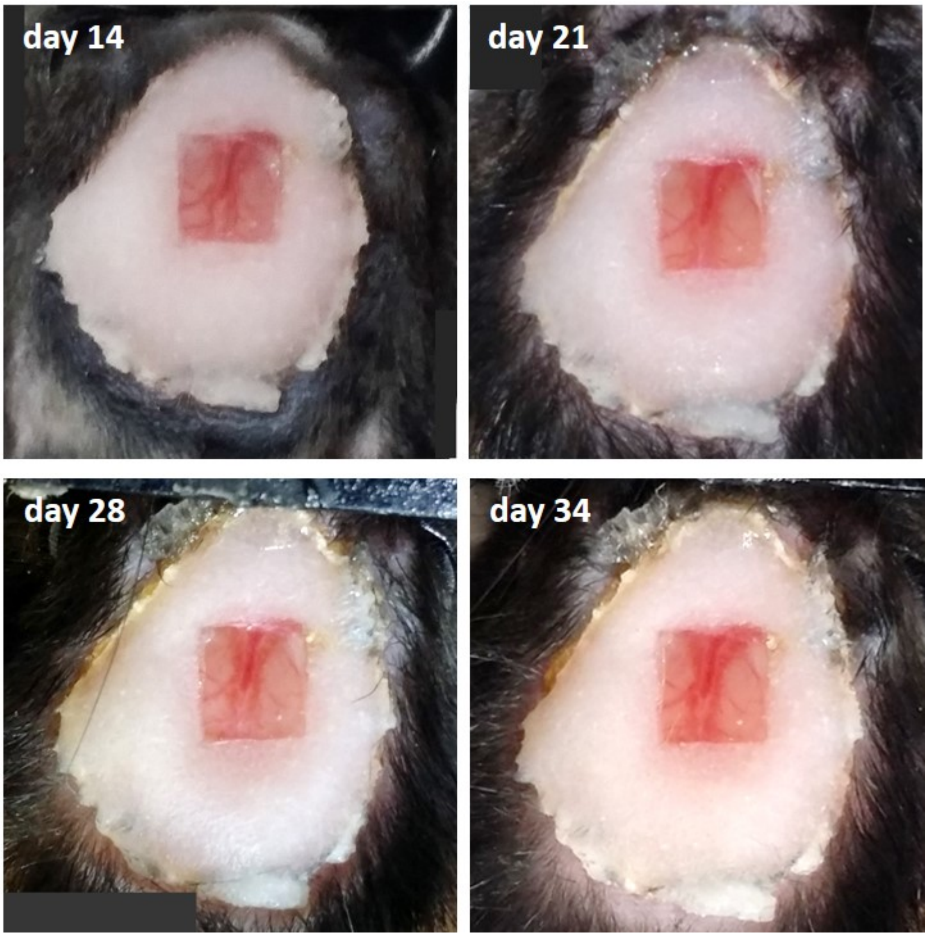

Supplementary Figure 1. Photographs of a calvarial window at different time points up to 34 days post window attachment. The calvaria and large calvarial blood vessels were clearly visible under the glass window at all the time points. There was no indication of scar formation.

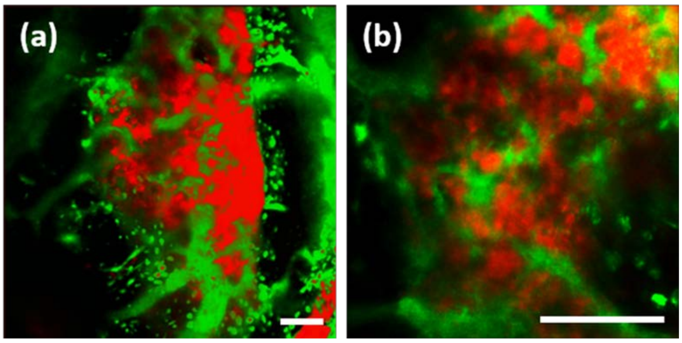

Supplementary Figure 2. Longitudinal TP images of the same BM cavities on day 28 post BMT. (a) MIP TP images in a large FOV, (b) single-plane TP images in a small FOV 110 μm below the bone surface. Blue: bone (SHG signal); red: transplanted BM cells (DsRed signal); green: vasculature (FITC-dextran signal). Scale bar: 100 μm.

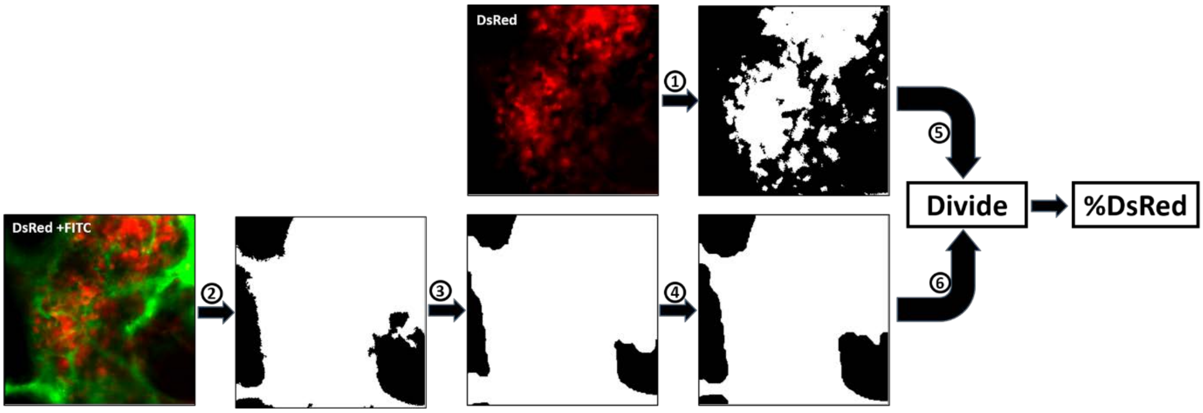

Supplementary Figure 3: Algorithm to calculate the percentage of DsRed-expressing (in pixels). (1) Thresholding, (2) thresholding and empty space filling, (3) structure dilating, (4) structural eroding, (5) (6) pixel counting.

Supplementary Video Legends

For the supplementary videos, the image size consists of 512 × 512 pixels covering a FOV of 775 μm × 775 μm for large FOV and 258 μm × 258 μm for small FOV with a step-wise increment of 2 μm along the z-axis for volumetric TP images. Imaging speed was 0.2 fps and video playback rate was 5 fps for volumetric TP videos and 8 fps for time-lapse TP videos. Color coding for fluorescent signals was as following: 430-480 nm (blue), 500-550 nm (green), and 625-675 nm (red).

**Supplementary Video 1**	3D volumetric TP video of a large FOV site of normal DsRed mouse calvarial bone marrow cavity. (Corresponding to Figure 2(a)).
**Supplementary Video 2**	3D volumetric TP video of a small FOV site of normal DsRed mouse calvarial bone marrow cavity. (Corresponding to Figure 2(b,c)).
**Supplementary Video 3**	Time-lapse TP video of a small FOV site of normal DsRed mouse calvarial bone marrow cavity.
**Supplementary Video 4**	Day 1: 3D volumetric TP video of the large FOV site of BM transplanted mouse calvarial bone marrow cavity. (Corresponding to Figure 3(a)).
**Supplementary Video 5**	Day 4: 3D volumetric TP video of the large FOV site of BM transplanted mouse calvarial bone marrow cavity. (Corresponding to Figure 3(b)).
**Supplementary Video 6**	Day 9: 3D volumetric TP video of the large FOV site of BM transplanted mouse calvarial bone marrow cavity. (Corresponding to Figure 3(c)).
**Supplementary Video 7**	Day 20: 3D volumetric TP video of the large FOV site of BM transplanted mouse calvarial bone marrow cavity. (Corresponding to Figure 3(d)).
**Supplementary Video 8**	Day 1: 3D volumetric TP video of the small FOV site of BM transplanted mouse calvarial bone marrow cavity. (Corresponding to Figure 3(e)).

**Supplementary Video 9**	Day 4: 3D volumetric TP video of the small FOV site of BM transplanted mouse calvarial bone marrow cavity. (Corresponding to Figure 3(f)).
**Supplementary Video 10**	Day 9: 3D volumetric TP video of the small FOV site of BM transplanted mouse calvarial bone marrow cavity. (Corresponding to Figure 3(g)).
**Supplementary Video 11**	Day 20: 3D volumetric TP video of the small FOV site of BM transplanted mouse calvarial bone marrow cavity. (Corresponding to Figure 3(h)).
**Supplementary Video 12**	Time-lapse TP video of the small FOV site of BM transplanted mouse calvarial bone marrow cavity on day 1.
**Supplementary Video 13**	Time-lapse TP video of a small FOV site of BM transplanted mouse calvarial bone marrow cavity on day 4. (Corresponding to Figure 4(a)).
**Supplementary Video 14**	Time-lapse TP video of a small FOV site of BM transplanted mouse calvarial bone marrow cavity on day 9. (Corresponding to Figure 4(b)).
**Supplementary Video 15**	Time-lapse TP video of a small FOV site of BM transplanted mouse calvarial bone marrow cavity on day 13. (Corresponding to Figure 4(c)).
